# Supplementary material for: Process evaluation of a randomised controlled trial aimed at improving health behaviours and vitamin D status during pregnancy: Implementation of the SPRING trial
Source: PLoS One. 2025 Sep 15;20(9):e0319224. doi: 10.1371/journal.pone.0319224 (PMC12435722; doi:10.1371/journal.pone.0319224)
Supplement: S1 Fig — (DOCX) [file pone.0319224.s001.docx]

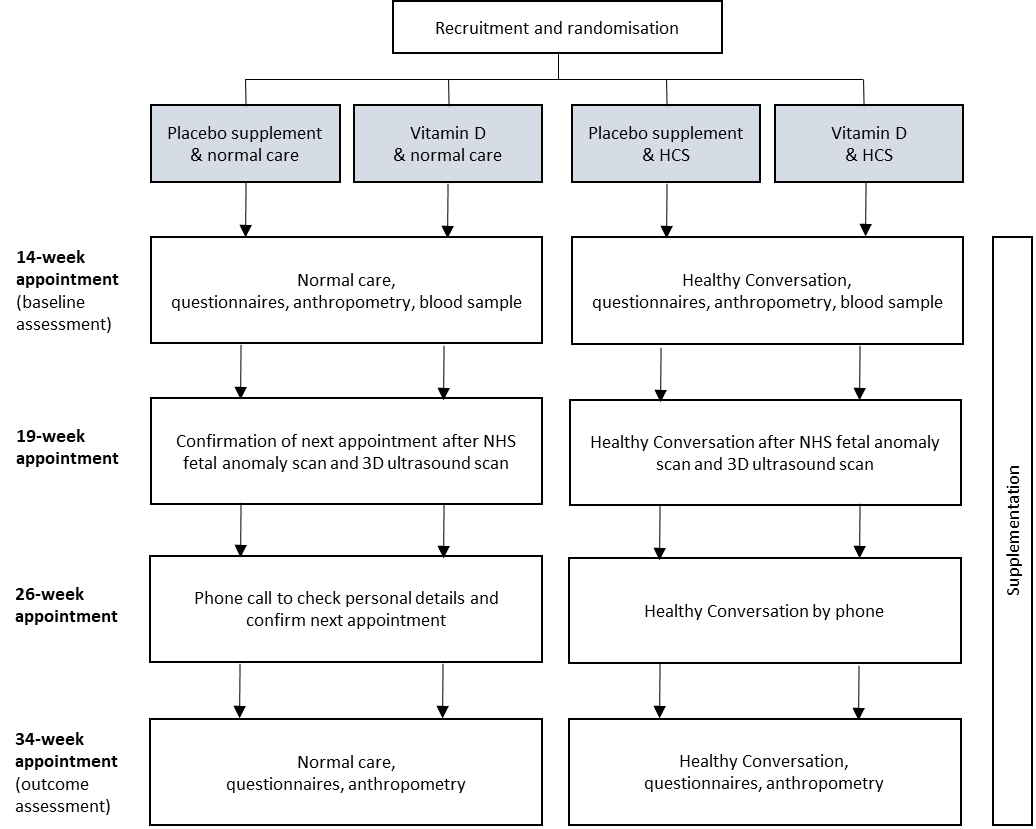


***S1 Fig****. Overview of the study design. HCS = Healthy Conversation Skills; NHS = National Health Service.*
