# Supplementary material for: Process evaluation of a randomised controlled trial aimed at improving health behaviours and vitamin D status during pregnancy: Implementation of the SPRING trial
Source: PLoS One. 2025 Sep 15;20(9):e0319224. doi: 10.1371/journal.pone.0319224 (PMC12435722; doi:10.1371/journal.pone.0319224)
Supplement: S3 Fig — (DOCX) [file pone.0319224.s003.docx]

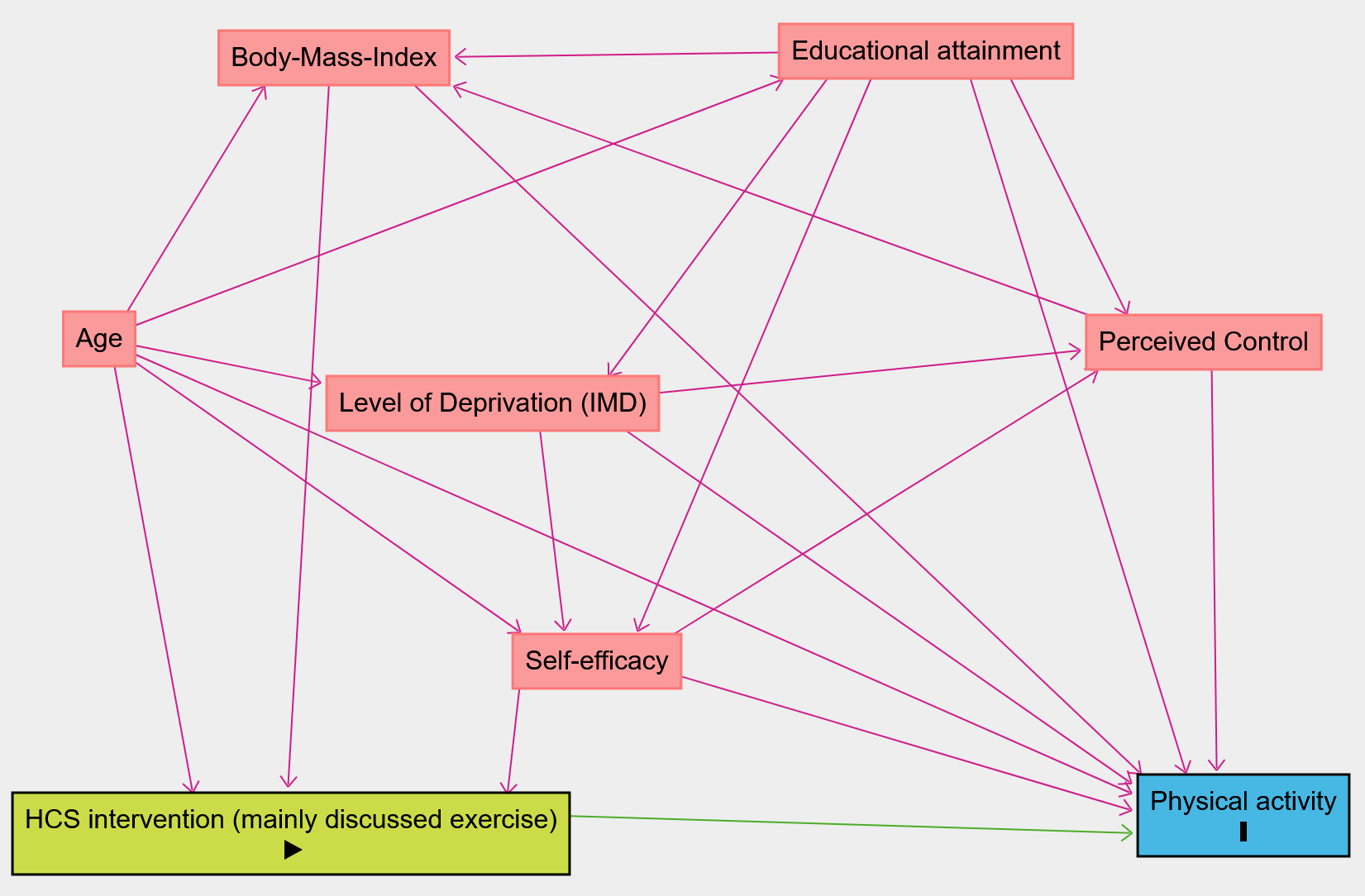


***S3 Fig****. Directed acyclic graph used to inform the model estimating the total effect of the Healthy Conversation Skills (HCS) intervention on physical activity among women who discussed physical activity as the main health behaviour. Green boxes indicate the exposure or an ancestor of the exposure, blue boxes indicate the outcome or an ancestor of the outcome, and pink boxes indicate ancestors of the exposure and outcome (confounders). The green arrow indicates the causal path and pink arrows are biasing paths. IMD, Index of Multiple Deprivation.*
