# Supplementary figures and images for: Process evaluation of a randomised controlled trial aimed at improving health behaviours and vitamin D status during pregnancy: Implementation of the SPRING trial
Source: PLoS One. 2025 Sep 15;20(9):e0319224. doi: 10.1371/journal.pone.0319224 (PMC12435722; doi:10.1371/journal.pone.0319224)

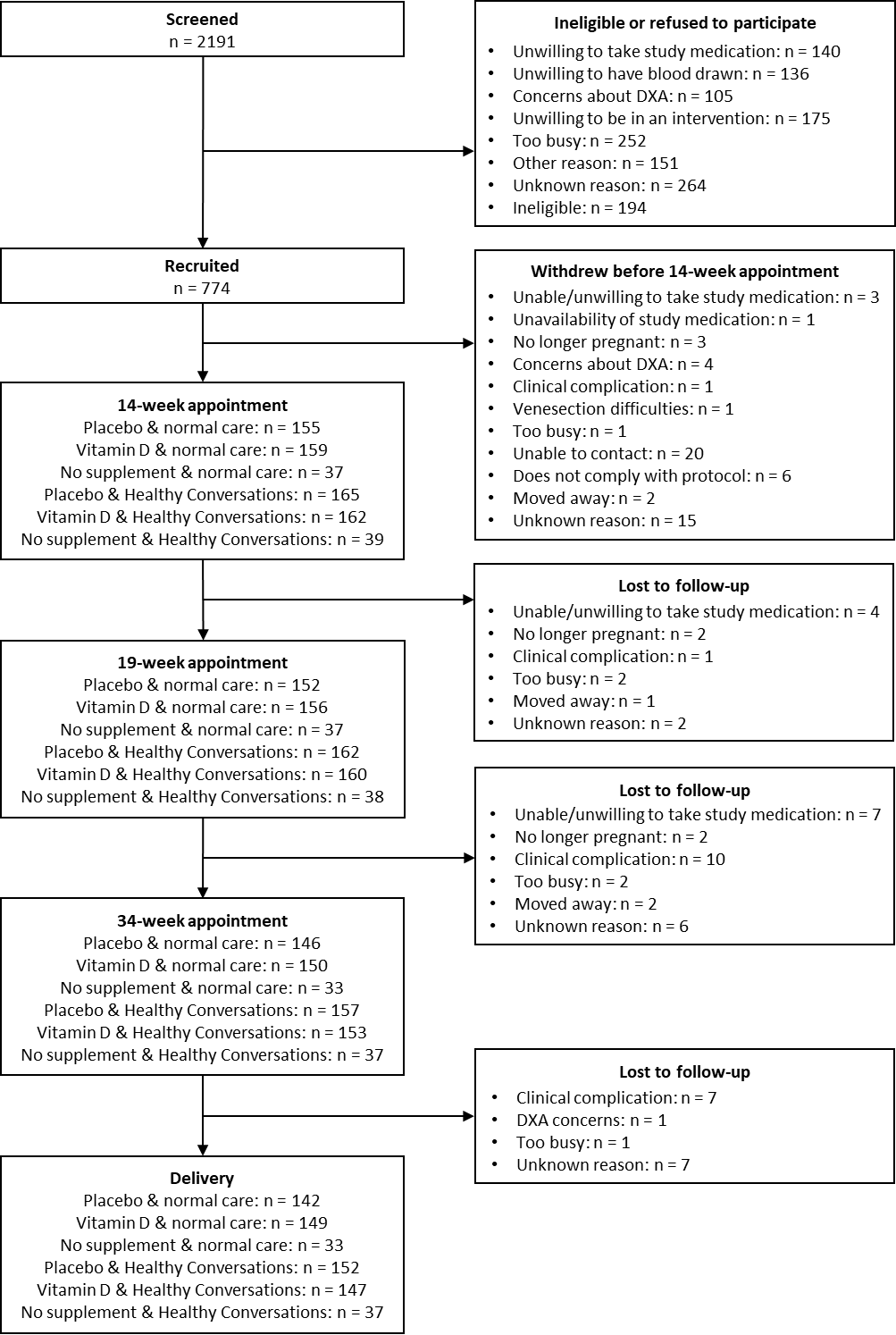


***S4 Fig****. Participant flow diagram.*

Supplement: S4 Fig — (DOCX) [file pone.0319224.s004.docx]
