# Supplementary material for: Process evaluation of a randomised controlled trial aimed at improving health behaviours and vitamin D status during pregnancy: Implementation of the SPRING trial
Source: PLoS One. 2025 Sep 15;20(9):e0319224. doi: 10.1371/journal.pone.0319224 (PMC12435722; doi:10.1371/journal.pone.0319224)
Supplement: S5 Fig — (DOCX) [file pone.0319224.s005.docx]

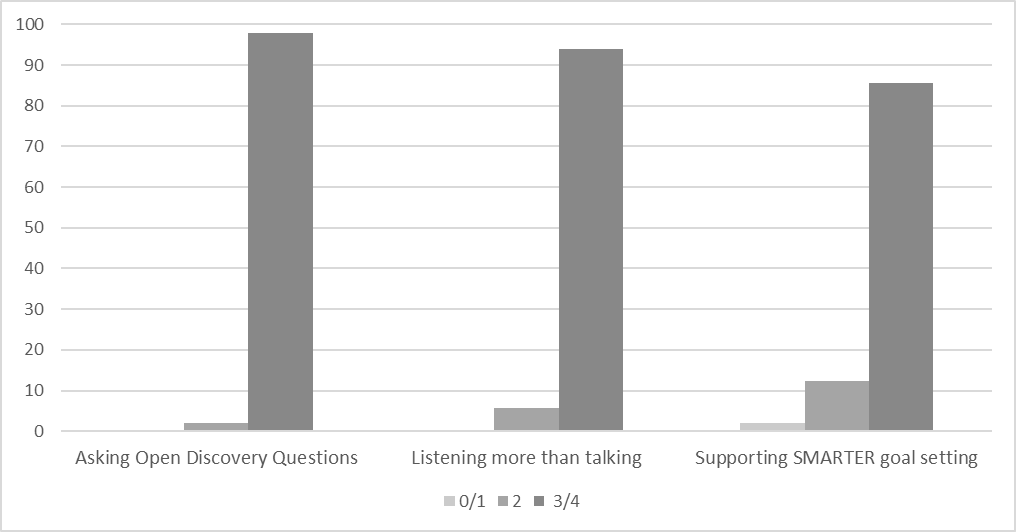


***S5 Fig****. Trained research nurses’ competency scores in using three Healthy Conversation Skills at the 26-week phone call. A score of 0 or 1 indicates low competence, 2 indicates medium competence, and 3 or 4 indicates high competence.*
