# Supplementary material for: Process evaluation of a randomised controlled trial aimed at improving health behaviours and vitamin D status during pregnancy: Implementation of the SPRING trial
Source: PLoS One. 2025 Sep 15;20(9):e0319224. doi: 10.1371/journal.pone.0319224 (PMC12435722; doi:10.1371/journal.pone.0319224)
Supplement: S1 Table — (DOCX) [file pone.0319224.s006.docx]

***S1 Table****. Data collection and processing of participants’ characteristics and health behaviours.*

| Measure | Method |
| --- | --- |
| Ethnicity | Participants were asked to indicate which ethnic group they felt they belonged to. Black Caribbean, Black African, and other Black group were combined into “Black”. Indian, Pakistani, Bangladeshi, Chinese, and other Asian group were grouped into “Asian”. Other options were “White” and “Other”. |
| Employment status | Women reported whether they were in paid employment or self-employed in the last year (yes/no). |
| Educational attainment | Educational attainment was categorised into three groups according to the women’s highest completed level of training. None, CSE, and O level/ GCSE were classified as “Low”, A level and HND as “Medium”, and Degree as “High”. |
| Level of deprivation | Level of deprivation, a measure of socioeconomic status, was assessed using the Index of Multiple Deprivation 2019 (IMD) (1). The IMD score is a relative measure, ranking small areas across England according to seven, weighted domains of deprivation (income, employment, education, health, crime, housing and services, and living environment). The overall score ranges from 1 (most deprived 10%) to 10 (least deprived 10%). Scores were calculated based on home postcodes using an online tool (2). |
| Self-efficacy | Self-efficacy was measured using a modified version of Schwarzer’s General Self-Efficacy Scale (3). The scale quantifies the belief in one’s ability to accomplish a task and overcome impediments. It is made up of five statements (e.g., ‘I can always manage to solve difficult problems if I try hard enough’), with possible responses being strongly disagree, disagree, agree, and strongly agree. These were coded from one (strongly disagree) to four (strongly agree) and summed up to give an overall score, ranging from 5 to 20, with higher scores indicating greater self-efficacy. |
| Perceived control | Women’s perceived control over life was assessed using a measure derived by Bobak et al. (4). Participants were asked to indicate whether they strongly disagree, disagree, agree, or strongly agree with five statements that indicate feeling in control (e.g., ‘Keeping healthy depends on things that I can do’) and four statements that indicate feeling out of control (e.g., ‘I feel that what happens in my life is often determined by factors beyond my control’). Responses were coded from one (strongly disagree) to four (strongly agree), with the scores reversed for items indicating feeling out of control. An overall score was calculated by summing up responses to each item. A higher overall score indicates a higher level of perceived control (range from 4 to 36). |
| Diet quality | Diet quality was assessed using a 20-item Food Frequency Questionnaire (FFQ) that was developed to measure adherence to a prudent dietary pattern among young women in Southampton (5). Participants indicated how often they consumed each of the 20 food items over the past three months, with possible answers being not in the past three months, once per month or less, once every two weeks, 1-2 times per week, 3-6 times per week, once a day, and more than once a day. For each food item, frequency of consumption was multiplied by the respective coefficient produced by principal component analysis. Results were added up and standardised to obtain an overall prudent diet score. |
| Physical activity | Physical activity was measured by asking participants to specify how much time they spent doing gentle (e.g., walking, gardening), moderate (e.g., easy swimming or cycling), and strenuous exercise (e.g., jogging, vigorous swimming or cycling) on average over the last three months. Answers to each category were summed to produce an overall measure of the average amount of hours per week each woman spent being physically active. Due to the high skewness of the physical activity data, the variable was transformed to create a Fisher-Yates normal score. |
| Alcohol consumption | Participants were asked to report how often and how many glasses/measures they drank of five different categories of alcoholic beverages over the past three months. These data were used to calculate the units of alcohol consumed by each woman on average per week. |
| Smoking status | Women self-reported if they were smoking at the time the questionnaire was administered (yes/no). |
| Problems with taking the study medication | The Problematic Experiences of Therapy Scale (PETS) (6) was used to assess participants’ perception of the extent to which they faced problems with taking the study medication. Three of the original four sub-scales were used: uncertainty about medication, doubts about medication, and practical problems. The fourth sub-scale relating to how symptoms hindered taking the study medication was not applicable. Each sub-scale encompassed a set of statements, for example, ‘I found it difficult to remember to take my capsules’ (practical problem). Possible responses were strongly disagree, disagree, agree, and strongly agree. Each sub-scale was dichotomised into “no problems” (women who responded with disagree or strongly disagree to each statement of the sub-scale) and “at least one problem” (women who responded with agree or strongly agree to at least one of the statements within the sub-scale). |

CSE, Certificate of Secondary Education; GCSE, General Certificate of Secondary Education; HND, Higher National Diploma.

**References**

1. Ministry of Housing Communities & Local Government. English indices of deprivation 2019 2019 [26 May 2023]. Available from: <https://www.gov.uk/government/statistics/english-indices-of-deprivation-2019>.

2. Ministry of Housing Communities & Local Government. English indices of deprivation 2019. Postcode Lookup 2019 [14 June 2023]. Available from: <https://imd-by-postcode.opendatacommunities.org/imd/2019>.

3. Luszczynska A, Scholz U, Schwarzer R. The general self-efficacy scale: multicultural validation studies. The Journal of psychology. 2005;139(5):439-57.

4. Bobak M, Pikhart H, Rose R, Hertzman C, Marmot M. Socioeconomic factors, material inequalities, and perceived control in self-rated health: cross-sectional data from seven post-communist countries. Soc Sci Med. 2000;51(9):1343-50.

5. Crozier SR, Inskip HM, Barker ME, Lawrence WT, Cooper C, Robinson SM. Development of a 20-item food frequency questionnaire to assess a 'prudent' dietary pattern among young women in Southampton. Eur J Clin Nutr. 2010;64(1):99-104.

6. Kirby S, Donovan-Hall M, Yardley L. Measuring barriers to adherence: validation of the Problematic Experiences of Therapy Scale. Disabil Rehabil. 2014;36(22):1924-9.
