# Supplementary material for: Process evaluation of a randomised controlled trial aimed at improving health behaviours and vitamin D status during pregnancy: Implementation of the SPRING trial
Source: PLoS One. 2025 Sep 15;20(9):e0319224. doi: 10.1371/journal.pone.0319224 (PMC12435722; doi:10.1371/journal.pone.0319224)
Supplement: S3 Table — (DOCX) [file pone.0319224.s008.docx]

***S3 Table.*** *Health behaviour discussed in the second most detail at each Healthy Conversation*

*(n, %).*

|  | 14 weeks | 19 weeks | 26 weeks | 34 weeks |
| --- | --- | --- | --- | --- |
| Diet | 180 (56.3) | 155 (46.1) | 157 (48.9) | 119 (36.6) |
| Physical Activity | 48 (15.0) | 36 (10.7) | 42 (13.1) | 49 (15.1) |
| Smoking | 22 (6.9) | 21 (6.3) | 22 (6.9) | 16 (4.9) |
| Alcohol | 1 (0.3) | 1 (0.3) | 0 | 1 (0.3) |
| Breastfeeding | 4 (1.3) | 72 (21.4) | 40 (12.5) | 110 (33.8) |
| Study Medication | 53 (16.6) | 28 (8.3) | 43 (13.4) | 19 (5.8) |
| Other | 12 (3.8) | 23 (6.8) | 17 (5.3) | 11 (3.4) |
