# Supplementary material for: Process evaluation of a randomised controlled trial aimed at improving health behaviours and vitamin D status during pregnancy: Implementation of the SPRING trial
Source: PLoS One. 2025 Sep 15;20(9):e0319224. doi: 10.1371/journal.pone.0319224 (PMC12435722; doi:10.1371/journal.pone.0319224)
Supplement: S4 Table — (DOCX) [file pone.0319224.s009.docx]

***S4 Table****. Association between exposure to the Healthy Conversation Skills intervention and diet quality at 34 weeks of gestation in subgroups of women who mainly discussed diet (SD scores).*

|  | Adjusted* | | Crude† | |
| --- | --- | --- | --- | --- |
|  | **β (95%-CI)** | **p value** | **β (95%-CI)** | **p value** |
| 14-week appointment | | | | |
| Control | ref |  | ref |  |
| Intervention (n=80) | -0.06 (-0.23; 0.12) | 0.5 | -0.05 (-0.23; 0.13) | 0.6 |
|  | **Adjusted R^2^** = 58.1 % |  | **Adjusted R^2^** = 55.0 % |  |
| 19-week appointment | | | | |
| Control | ref |  | ref |  |
| Intervention (n=55) | -0.02 (-0.23; 0.19) | 0.8 | -0.00 (-0.21; 0.21) | >0.9 |
|  | **Adjusted R^2^** = 59.7 % |  | **Adjusted R^2^** = 56.9 % |  |
| 26-week phone call | | | | |
| Control | ref |  | ref |  |
| Intervention (n=59) | -0.10 (-0.30; 0.10) | 0.3 | -0.08 (-0.28; 0.12) | 0.4 |
|  | **Adjusted R^2^** = 58.6 % |  | **Adjusted R^2^** = 54.7 % |  |

* Adjusted for dietary quality score at baseline, age, educational attainment, Index of Multiple Deprivation, perceived control, and self-efficacy. † Adjusted for dietary quality score at baseline. CI, confidence interval.
