# Supplementary material for: Process evaluation of a randomised controlled trial aimed at improving health behaviours and vitamin D status during pregnancy: Implementation of the SPRING trial
Source: PLoS One. 2025 Sep 15;20(9):e0319224. doi: 10.1371/journal.pone.0319224 (PMC12435722; doi:10.1371/journal.pone.0319224)
Supplement: S6 Table — (DOCX) [file pone.0319224.s011.docx]

***S6 Table****. Baseline characteristics of participants in the control group, those in the intervention group that discussed physical activity as the main health behaviour (included in the subgroup analysis) and those in the intervention group that did not discuss physical activity as the main health behaviour (excluded from the subgroup analysis).*

|  |  | Control  n = 351 | Intervention & primarily discussed physical activity at least once  n = 316 | Intervention & never discussed physical activity as the primary health behaviour  n = 50 | p value^a^ |
| --- | --- | --- | --- | --- | --- |
| **Age [years]**, mean ± SD |  | 31.3 ± 4.9 | 31.7 ± 5.1 | 29.1 ± 5.8 | 0.3 |
| **Ethnicity**, n, % | White  Other | 332 (94.6)  19 (5.4) | 292 (92.7)  23 (7.3) | 48 (96.0)  2 (4.0) | 0.3 |
| **Deprivation**, median (IQR) | Index of Multiple Deprivation | 6 (4; 8) | 6 (4; 8) | 5 (2; 8) | 0.6 |
| **Educational attainment**, n (%) | Low (None, CSE, O levels)  Medium (A levels, HND)  High (Degree) | 48 (13.7)  122 (34.9)  180 (51.4) | 43 (13.8)  96 (30.8)  173 (55.5) | 20 (40.0)  19 (38.0)  11 (22.0) | 0.5 |
| **Number of children**, n (%) | 0  1  2  More than 3 | 141 (40.8)  145 (41.9)  45 (13.0)  15 (4.3) | 133 (42.4)  123 (39.2)  46 (14.7)  12 (3.8) | 19 (38.0)  19 (38.0)  6 (12.0)  6 (12.0) | 0.8 |
| **Weight**, n (%) | Underweight  Normal weight  Overweight  Obesity | 9 (2.6)  154 (44.5)  119 (34.4)  64 (18.5) | 3 (1.0)  124 (39.5)  112 (35.7)  75 (23.9) | 1 (2.0)  18 (36.0)  11 (22.0)  20 (40.0) | 0.1^b^ |
| **Diet Quality**, mean ± SD | Dietary Quality Score | 0.09 ± 1.03 | 0.01 ± 0.95 | -0.65 ± 0.90 | 0.3 |
| **Physical Activity**, median (IQR) | Hours spent being physically active per week | 2.0 (1.3; 3.0) | 2.0 (1.3; 2.7) | 2.0 (1.0; 3.0) | 0.7 |

^a^ Comparison between participants in the control group and those in the intervention group who primarily discussed physical activity at least once. ^b^ The categories underweight and normal weight were merged before running a chi^2^ test. CSE, Certificate of Secondary Education; HND, Higher National Diploma.
