# Supplementary material for: Process evaluation of a randomised controlled trial aimed at improving health behaviours and vitamin D status during pregnancy: Implementation of the SPRING trial
Source: PLoS One. 2025 Sep 15;20(9):e0319224. doi: 10.1371/journal.pone.0319224 (PMC12435722; doi:10.1371/journal.pone.0319224)
Supplement: S7 Table — (DOCX) [file pone.0319224.s012.docx]

***S7 Table.*** *Baseline characteristics of participants in the control group and those in the intervention group who discussed physical activity as the main health behaviour never, once, twice and three times.*

|  |  | Control  n = 351 | Intervention & never discussed physical activity as the primary health behaviour  n = 50 | Intervention & discussed physical activity once as the primary health behaviour  n = 51 | Intervention & discussed physical activity twice as the primary health behaviour  n = 101 | Intervention & discussed physical activity three times as the primary health behaviour  n = 164 |
| --- | --- | --- | --- | --- | --- | --- |
| Age [years], mean ± SD |  | 31.3 ± 4.9 | 29.1 ± 5.8 | 29.6 ± 5.2 | 31.3 ± 5.7 | 32.6 ± 4.4 |
| Ethnicity, n, % | White  Other | 332 (94.6)  19 (5.4) | 48 (96.0)  2 (4.0) | 46 (92.0)  4 (8.0) | 96 (95.1)  5 (4.9) | 150 (91.5)  14 (8.5) |
| Deprivation, median (IQR) | Index of Multiple Deprivation | 6 (4; 8) | 5 (2; 8) | 5 (2; 7) | 6 (4; 8) | 6 (4; 8) |
| Educational attainment, n (%) | Low (None, CSE, O levels)  Medium (A levels, HND)  High (Degree) | 48 (13.7)  122 (34.9)  180 (51.4) | 20 (40.0)  19 (38.0)  11 (22.0) | 12 (24.5)  17 (34.7)  20 (40.8) | 11 (11.1)  37 (37.4)  51 (51.5) | 20 (12.2)  42 (25.6)  102 (62.2) |
| Number of children, n (%) | 0  1  2  More than 3 | 141 (40.8)  145 (41.9)  45 (13.0)  15 (4.3) | 19 (38.0)  19 (38.0)  6 (12.0)  6 (12.0) | 19 (38.8)  21 (42.9)  7 (14.3)  2 (4.1) | 34 (33.7)  47 (46.5)  16 (15.8)  4 (4.0) | 80 (48.8)  55 (33.5)  23 (14.0)  6 (3.7) |
| Weight, n (%) | Underweight  Normal weight  Overweight  Obesity | 9 (2.6)  154 (44.5)  119 (34.4)  64 (18.5) | 1 (2.0)  18 (36.0)  11 (22.0)  20 (40.0) | 1 (2.0)  16 (32.0)  21 (42.0)  12 (24.0) | 1 (1.0)  42 (42.0)  31 (31.0)  26 (26.0) | 1 (0.6)  66 (40.2)  60 (36.6)  37 (22.6) |
| Diet Quality, mean ± SD | Dietary Quality Score | 0.09 ± 1.03 | -0.65 ± 0.90 | -0.56 ± 1.03 | -0.09 ± 0.84 | 0.24 ± 0.90 |
| Physical Activity, median (IQR) | Hours spent being physically active per week | 2.0 (1.3; 3.0) | 2.0 (1.0; 3.0) | 1.9 (1.2; 3.0) | 2.0 (1.3; 2.8) | 2.0 (1.5; 2.5) |

CSE, Certificate of Secondary Education; HND, Higher National Diploma.
