# Supplementary material for: Process evaluation of a randomised controlled trial aimed at improving health behaviours and vitamin D status during pregnancy: Implementation of the SPRING trial
Source: PLoS One. 2025 Sep 15;20(9):e0319224. doi: 10.1371/journal.pone.0319224 (PMC12435722; doi:10.1371/journal.pone.0319224)
Supplement: Attachment — Submitted filename: PLOS ONE_Response to Queries_Rebuttal Letter 210725final.docx [file pone.0319224.s014.docx]

Francis Xavier Kasujja
Academic Editor

PLOS ONE

21st July 2025

Dear Dr Kasujja,

Thank you for the opportunity to revise and resubmit our manuscript entitled **“Process evaluation of a randomised controlled trial aimed at improving health behaviours and vitamin D status during pregnancy: Implementation of the SPRING trial”**.

We would like to thank the editor and reviewers for their careful evaluation of our manuscript and their insightful feedback. We have carefully addressed all comments and revised our manuscript accordingly. We believe that this helped improve the quality of our work.

Below, we provide a detailed response to each of the comments. Where applicable, we have noted the specific changes made to the manuscript. We hope that the revisions satisfactorily address the concerns raised and that the manuscript is now suitable for publication in PLOS ONE.

**Response to the Editor**

1. Please ensure that your manuscript meets PLOS ONE's style requirements, including those for file naming.

Author’s reply: We have made sure that our manuscript adheres to PLOS ONE's style requirements.

2. Thank you for stating in your Funding Statement: […] For the purpose of Open Access, the author has applied a Creative Commons Attribution (CC BY) licence to any Author Accepted Manuscript version arising from this submission.”

Please provide an amended statement that declares *all* the funding or sources of support (whether external or internal to your organization) received during this study, as detailed online in our guide for authors at http://journals.plos.org/plosone/s/submit-now. Please also include the statement “There was no additional external funding received for this study.” in your updated Funding Statement.

Please include your amended Funding Statement within your cover letter. We will change the online submission form on your behalf.

Author’s reply: We have updated the Funding Statement accordingly. Please find the revised Funding Statement at the end of this section.

3. Thank you for stating the following in the Competing Interests section: […] We note that one or more of the authors are employed by a commercial company

a. Please provide an amended Funding Statement declaring this commercial affiliation, as well as a statement regarding the Role of Funders in your study. If the funding organization did not play a role in the study design, data collection and analysis, decision to publish, or preparation of the manuscript and only provided financial support in the form of authors' salaries and/or research materials, please review your statements relating to the author contributions, and ensure you have specifically and accurately indicated the role(s) that these authors had in your study. You can update author roles in the Author Contributions section of the online submission form.

Please also include the following statement within your amended Funding Statement.

“The funder provided support in the form of salaries for authors [insert relevant initials], but did not have any additional role in the study design, data collection and analysis, decision to publish, or preparation of the manuscript. The specific roles of these authors are articulated in the ‘author contributions’ section.”

If your commercial affiliation did play a role in your study, please state and explain this role within your updated Funding Statement.

Author’s reply: While Professors Harvey and Cooper have carried out consultancy work, as indicated in our previous statement, neither of them is employed by these companies. So, we are unclear why you have suggested they are employed by a commercial company. The facts we have stated are correct in relation to their competing interests. We have added that their consultancy work did not play a role in this study. We trust that this addresses your comments.

b. Please also provide an updated Competing Interests Statement declaring this commercial affiliation along with any other relevant declarations relating to employment, consultancy, patents, products in development, or marketed products, etc.

Within your Competing Interests Statement, please confirm that this commercial affiliation does not alter your adherence to all PLOS ONE policies on sharing data and materials by including the following statement: "This does not alter our adherence to PLOS ONE policies on sharing data and materials.” (as detailed online in our guide for authors http://journals.plos.org/plosone/s/competing-interests) . If this adherence statement is not accurate and there are restrictions on sharing of data and/or materials, please state these. Please note that we cannot proceed with consideration of your article until this information has been declared.

Please include both an updated Funding Statement and Competing Interests Statement in your cover letter. We will change the online submission form on your behalf.

Author’s reply: We have added the statement about PLOS ONE policies on sharing data and materials to our competing interests statement.

4. We note that you have indicated that there are restrictions to data sharing for this study. For studies involving human research participant data or other sensitive data, we encourage authors to share de-identified or anonymized data. However, when data cannot be publicly shared for ethical reasons, we allow authors to make their data sets available upon request. For information on unacceptable data access restrictions, please see http://journals.plos.org/plosone/s/data-availability#loc-unacceptable-data-access-restrictions.

Before we proceed with your manuscript, please address the following prompts:

a) If there are ethical or legal restrictions on sharing a de-identified data set, please explain them in detail (e.g., data contain potentially identifying or sensitive patient information, data are owned by a third-party organization, etc.) and who has imposed them (e.g., a Research Ethics Committee or Institutional Review Board, etc.). Please also provide contact information for a data access committee, ethics committee, or other institutional body to which data requests may be sent.

b) If there are no restrictions, please upload the minimal anonymized data set necessary to replicate your study findings to a stable, public repository and provide us with the relevant URLs, DOIs, or accession numbers. Please see http://www.bmj.com/content/340/bmj.c181.long for guidelines on how to de-identify and prepare clinical data for publication. For a list of recommended repositories, please see https://journals.plos.org/plosone/s/recommended-repositories. You also have the option of uploading the data as Supporting Information files, but we would recommend depositing data directly to a data repository if possible.

Please update your Data Availability statement in the submission form accordingly.

Author’s reply: We have reviewed our ethics and governance arrangements for the study and, as a result, we confirm that we will make our data available in the University of Southampton open access repository (PURE https://pure.soton.ac.uk/).

5. In the online submission form, you indicated that your data is available only on request from a third party. Please note that your Data Availability Statement is currently missing [the name of the third party contact or institution / contact details for the third party, such as an email address or a link to where data requests can be made]. Please update your statement with the missing information.

Author’s reply: Due to the updates made to our Data Availability Statement, this is no longer applicable.

Please find below the updated Funding and Competing Interests Statements.

Funding Statement

This work was supported by the UK Medical Research Council [MC_PC_21003; MC_PC_21001] and National Institute for Health and Care Research National Institute for Health Research (NIHR) Southampton Biomedical Research Centre, University of Southampton and University Hospital Southampton NHS Foundation Trust. The work leading to these results was supported by the European Union’s Seventh Framework Programme (FP7/2007–2013), projects EarlyNutrition, ODIN and LifeCycle under grant agreements numbers 289346, 613977 and 733206, and by the BBSRC (HDHL-Biomarkers, BB/P028179/1 and BB/P028187/1), as part of the ALPHABET project, supported by an award made through the ERA-Net on Biomarkers for Nutrition and Health (ERA HDHL), Horizon 2020 grant agreement number 696295. We are extremely grateful to Merck GmbH for the kind provision of the Vigantoletten supplement. The funders had no role in study design, data collection and analysis, decision to publish, or preparation of the manuscript. There was no additional external funding received for this study.

The funders (UK MRC, NIHR Southampton BRC, University of Southampton and University Hospital Southampton NHS Foundation Trust) provided support in the form of salaries for authors [NCH, JB, MEB, CC, MB, WTL, CV, SS, HMI, JH, KM, KH] but did not have any additional role in the study design, data collection and analysis, decision to publish, or preparation of the manuscript. The specific roles of these authors are articulated in the ‘author contributions’ section.

For the purpose of Open Access, the author has applied a Creative Commons Attribution (CC BY) licence to any Author Accepted Manuscript version arising from this submission.

Competing Interests Statement

Janis Baird, Mary Barker and Wendy Lawrence have received grant research support from Danone Nutricia Early Nutrition. Cyrus Cooper has received consultancy, lecture fees and honoraria from AMGEN, GSK, Alliance for Better Bone Health, MSD, Eli Lilly, Pfizer, Novartis, Servier, Merck, Medtronic and Roche. Members of Hazel Inskip’s team have received grant research support from Nestec and Danone Nutricia Early Life Nutrition. Nicholas Harvey has received consultancy, lecture fees and honoraria from Alliance for Better Bone Health, AMGEN, MSD, Eli Lilly, Servier, Shire, UCB, Consilient Healthcare, Theramex, Kyowa Kirin and Internis Pharma. The commercial companies that Professors Harvey and Cooper undertook consultancy work for did not play any role in this study. Professors Havey and Cooper were not directly employed by these companies. None of the interests declared alter our adherence to PLOS ONE policies on sharing data and materials.

**Response to the Reviewers**

Reviewer #1

Differences between groups were assessed using normal univariate statistics for continuous data and non parametric for non normal data. For the subgroup analyses, multiple linear regression models were used to compare the intervention and control groups. The R square values on the multiple regression might be helpful.

Author’s reply: We have added the R squared values to Tables 8 and S4 as requested. However, we feel that this makes the tables rather complicated to read and we will leave it to the editor’s discretion to decide if this change should be kept.

To account for differences between the groups at baseline, analyses were adjusted for potential confounders. The analysis as discussed in the statistical section was routine as one would expect in this type of application. The analysis was done appropriately but could have been improved. Also, the analysis was presented somewhat fragmented. There were many pieces to try to digest separately.

Author’s reply: We have reordered the results section to present our findings in a sequence that we hope improves clarity and coherence.

Groups were compared but at various times. What about a group or time effect overall? Was there any group by interaction effect?

Author’s reply: Our senior statistician has reviewed this and is not sure what is meant by a group by interaction effect. We believe that our analysis of data collected at the final 34-week time point, adjusted for the 14-week baseline measure, effectively takes account of group and time.

Figure S3 raises issues as to what is possibly the best intervention compared to control?

Author’s reply: Figure S3 is our CONSORT diagram. This diagram is designed to explain the flow of participants through the trial, not to compare intervention to control.

Fidelity, dose, and reach were evaluated descriptively. It is not really clear how much this added to the entire objectives of the study which mainly was to compare the vitamin D to the Healthy Conversation groupings. The design of the study was of the factorial nature. Sample size calculations were implemented.

Author’s reply: It was our aim to only investigate the interventions’ implementation - namely reach, fidelity, and dose - and to evaluate how variations in implementation influenced the effectiveness of the intervention. The trial outcomes will be published separately. We have added the following sentence to the Introduction section (ll. 92-93 in tracked changes version) for clarification:

*The results of the outcome evaluation will be published separately and will be informed by the findings of this process evaluation.*

One tries to get an idea of the flow of the study in a graphic and Figure S3 was not very helpful. A good schematic of the mechanics of the study showing what happens and when would have been more helpful.

Author’s reply: Figure S3 (now renamed to Figure S4) is a CONSORT diagram and adheres to the CONSORT statement. To address your comment, we added an overview of the study in the Supporting Information (S1 Fig) and added a reference to the additional figure in the Methods section under “The SPRING trial” (l. 113 in tracked changes version):

*An overview of the study design is presented in S1 Fig.*

The conclusion appears to dwell on intervention delivery and the mechanics of such. Where exactly is there a statement that states the women’s nutritional status was improved or not overall?

Author’s reply: As stated in our reply above, the focus of our manuscript is the evaluation of the intervention’s implementation, not an outcome assessment. We hope that the sentence added to the manuscript (see reply above) provides the necessary clarification for the reader.

Reviewer #2

The manuscript is clearly written and of high quality. The results are very valuable for contextualizing the study's efficacy findings and to understand the implementation, dose and reach of the intervention.

Introduction/Methods: Please provide brief background information on the Healthy Conversation Skills intervention, especially whether this intervention has been implemented and evaluated among pregnant women in other settings, and if so, what the general results were regarding its efficacy.

Author’s reply: As indicated in our response to Reviewer 1, this paper focuses on process and is not reporting outcomes. The outcome assessment will be published separately. Therefore, we have not added background information on the evaluation of the HCS intervention in the Introduction and Methods section. We have provided a brief overview of previous HCS assessments in the Discussion section (see ll. 583-598 in tracked changes version).

Intervention Line 131: Please clarify the context of Vitamin D supplementation among pregnant women in the study area. Are women in the study area advised by their health care providers to take their own Vitamin D supplementation or were they relying on it being given by the hospital? If the former, could some participants in both control and intervention groups have taken Vitamin D and could that have confounded the final trial's efficacy results?

Author’s reply: The trial assessed the effect of the prescription of Vitamin D delivered as part of the intervention. Some women in the study might have chosen to take Vitamin D themselves, consistent with public health advice, but our measurements at baseline showed little difference in Vitamin D levels between intervention and control.

Line 136: Participants were recruited at less than 17 weeks’ gestation. Participants recruited between 15-17 weeks would have missed their baseline appointment at 14 weeks. When did they receive their capsules?

Author’s reply: Participants who were recruited later than 14 weeks’ gestation were given their capsules as soon after recruitment as possible. We added a sentence to the Methods section under “Interventions” (ll. 141-142 in tracked changes version) to clarify this:

*Women recruited after 14 weeks’ gestation received the study medication at the earliest opportunity following recruitment.*

Line 154: same question as above for the Healthy Conversations with women at 14 weeks and the baseline interview at 14 weeks.

Author’s reply: Those participants who were recruited later than 14 weeks’ gestation had their first Healthy Conversation as soon after recruitment as possible. We added a sentence to the Methods section under “Interventions” (ll. 159-161 in tracked changes version) for clarification:

*Participants recruited after 14 weeks’ gestation had their first Healthy Conversation at the earliest opportunity following recruitment.*

Line 171: “Pill counts were conducted at the 19- and 34-week appointments..”: We assume pill counts were self-reported, please specify.

Author’s reply: We added details on this to the Methods section under “Data collection” (l. 177 in tracked changes version).

*Pill counts to assess compliance with taking the study medication were conducted at the 19- and 34-week appointments and at delivery. These were based on the blister packages brought to the visits.*

Results Line 320-322: Pregnant women were recruited from the main maternity hospital in Southhampton. The Reach results can be contextualised with respect to how representative the maternity hospital setting is of all pregnant women in Southhampton. Please clarify if some Southhampton women seek antenatal care at other types of health facilities other than this maternity hospital. This could contextualise why the SPRING sample included higher proportions of older women and lower proportions of younger women. Could it be that younger women access antenatal care from the maternity hospital at late stages in their pregnancy (beyond 17 weeks) or younger women and those from ethnic minorities access care from other types of facilities?

Author’s reply: The Princess Anne Hospital is the only public maternity hospital in Southampton and we believe that the lower proportion of younger women and those from ethnic minorities in the study reflect the challenges of recruitment. The reviewer is correct in that we would have missed women attending for their first antenatal appointment after 17 weeks’ gestation. We have included this additional explanation in our manuscript in the Discussion section under “Reach” (ll. 486-488 in tracked changes version):

*While a contributing factor might be the fact that women presenting for their first antenatal care appointment after 17 weeks’ gestation were missed, these findings most likely reflect the challenges of recruitment in these groups.*

The focus of the Healthy Conversation was determined by what each woman wanted to discuss as the primary health behaviour (e.g. physical activity/diet/other). Women with low educational attainment and obese women were more likely to never discus physical activity as the primary health behaviour. This could be due to lack of awareness/knowledge/health literacy on the importance of physical activity or perceived behavioural control, or lack of motivation, among other reasons. While not the focus of the study, could the authors recommend or comment on ways in which this intervention, or future interventions, could promote women (particularly those with low educational attainment or who are obese) to start talking about ways to improve their physical activity during pregnancy and to recognise the importance of physical activity.

Author’s reply: We added a paragraph to the Discussion section under “Fidelity” (ll. 546-554 in tracked changes version):

*At the same time, it is important to note that* *women of low education attainment and obese women were less likely to discuss physical activity during the Healthy Conversations. For women of low SES, lower levels of health literacy and limited resources might make physical activity a low priority (47).* *Women living with overweight and obesity may be concerned about exercising safely and also experience lack of time and opportunity to exercise (**48). In the future, these factors need to be addressed to ensure that the intervention reaches and resonates with all women, particularly those who may face greater barriers to engaging in physical activity during pregnancy.*

We hope that we have addressed all queries to your and the reviewer’s satisfaction.

Yours sincerely,

Simone Proebstl

Research Associate at LMU Munich

Former intern at the MRC Lifecourse Epidemiology Centre (during the preparation of the manuscript)

Janis Baird

Professor of Public Health and Epidemiology

MRC Lifecourse Epidemiology Centre

University of Southampton
